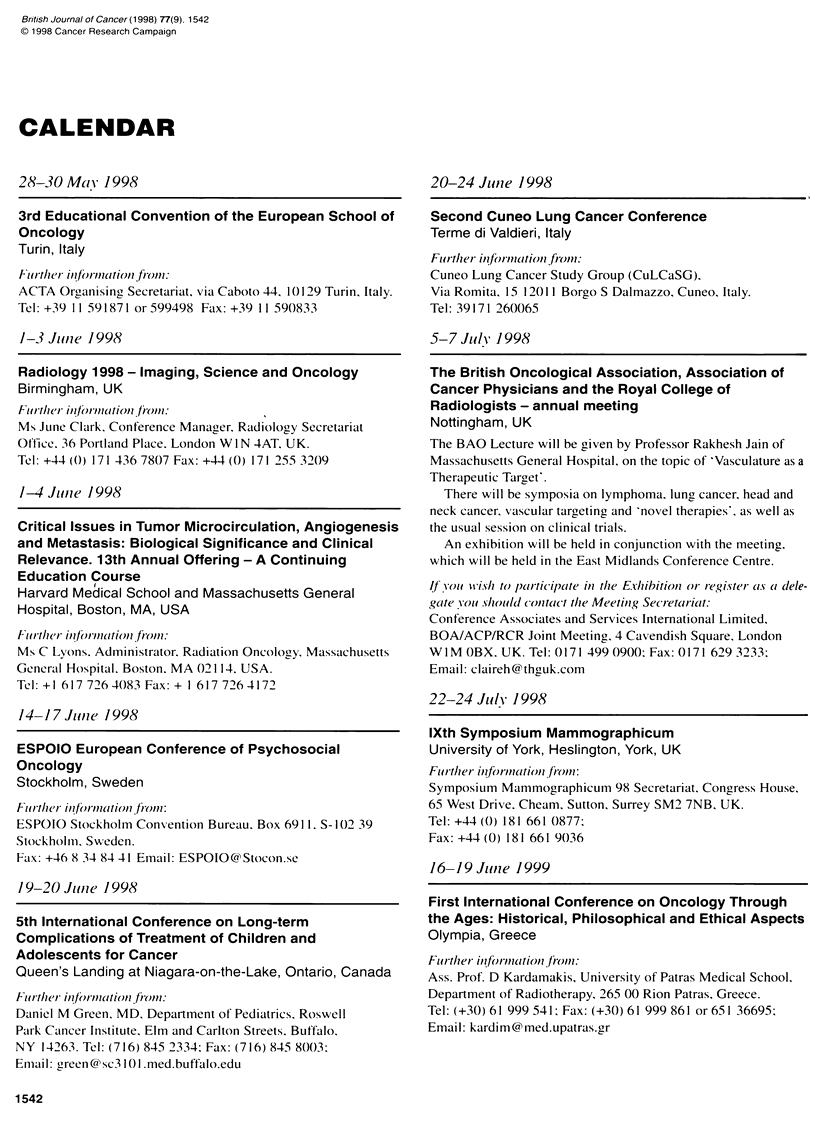# Calendar

**Published:** 1998-05

**Authors:** 


British Journal of Cancer (1998) 77(9), 1542
© 1998 Cancer Research Campaign

### CALENDAR

*28–30 May 1998*

**3rd Educational Convention of the European School of Oncology**
Turin, Italy

*Further information from:*

ACTA Organising Secretariat, via Caboto 44, 10129 Turin, Italy.

Tel: +39 11 591871 or 599498 Fax: +39 11 590833

*1–3 June 1998*

**Radiology 1998 – Imaging, Science and Oncology**
Birmingham, UK

*Further information from:*

Ms June Clark, Conference Manager, Radiology Secretariat Office, 36 Portland Place, London W1N 4AT, UK.

Tel: +44 (0) 171 436 7807 Fax: +44 (0) 171 255 3209

*1–4 June 1998*

**Critical Issues in Tumor Microcirculation, Angiogenesis and Metastasis: Biological Significance and Clinical Relevance. 13th Annual Offering – A Continuing Education Course**
Harvard Medical School and Massachusetts General Hospital, Boston, MA, USA

*Further information from:*
Ms C Lyons, Administrator, Radiation Oncology, Massachusetts General Hospital, Boston, MA 02114, USA.
Tel: +1 617 726 4083 Fax: + 1 617 726 4172

*14–17 June 1998*

**ESPOIO European Conference of Psychosocial Oncology**
Stockholm, Sweden

*Further information from:*

ESPOIO Stockholm Convention Bureau, Box 6911, S-102 39 Stockholm, Sweden.

Fax: +46 8 34 84 41 Email: ESPOIO@Stocon.se

*19–20 June 1998*

**5th International Conference on Long-term Complications of Treatment of Children and Adolescents for Cancer**
Queen's Landing at Niagara-on-the-Lake, Ontario, Canada

*Further information from:*
Daniel M Green, MD, Department of Pediatrics, Roswell Park Cancer Institute, Elm and Carlton Streets, Buffalo, NY 14263. Tel: (716) 845 2334; Fax: (716) 845 8003;
Email: green@sc3101.med.buffalo.edu

*20–24 June 1998*

**Second Cuneo Lung Cancer Conference**
Terme di Valdieri, Italy

*Further information from:*
Cuneo Lung Cancer Study Group (CuLCaSG),
Via Romita, 15 12011 Borgo S Dalmazzo, Cuneo, Italy.
Tel: 39171 260065

*5–7 July 1998*

**The British Oncological Association, Association of Cancer Physicians and the Royal College of Radiologists – annual meeting**
Nottingham, UK

The BAO Lecture will be given by Professor Rakhesh Jain of Massachusetts General Hospital, on the topic of 'Vasculature as a Therapeutic Target'.

There will be symposia on lymphoma, lung cancer, head and neck cancer, vascular targeting and 'novel therapies', as well as the usual session on clinical trials.

An exhibition will be held in conjunction with the meeting, which will be held in the East Midlands Conference Centre.

*If you wish to participate in the Exhibition or register as a delegate you should contact the Meeting Secretariat:*

Conference Associates and Services International Limited, BOA/ACP/RCR Joint Meeting, 4 Cavendish Square, London W1M 0BX, UK. Tel: 0171 499 0900; Fax: 0171 629 3233;

Email: claireh@thguk.com

*22–24 July 1998*

**IXth Symposium Mammographicum**
University of York, Heslington, York, UK

*Further information from:*
Symposium Mammographicum 98 Secretariat, Congress House, 65 West Drive, Cheam, Sutton, Surrey SM2 7NB, UK.
Tel: +44 (0) 181 661 0877;
Fax: +44 (0) 181 661 9036

*16–19 June 1999*

**First International Conference on Oncology Through the Ages: Historical, Philosophical and Ethical Aspects**
Olympia, Greece

*Further information from:*
Ass. Prof. D Kardamakis, University of Patras Medical School, Department of Radiotherapy, 265 00 Rion Patras, Greece.
Tel: (+30) 61 999 541; Fax: (+30) 61 999 861 or 651 36695;
Email: kardim@med.upatras.gr